# Inclusion of people with multiple long-term conditions in pregnancy research: patient, public and stakeholder involvement and engagement in a randomised controlled trial

**DOI:** 10.1186/s40900-024-00634-7

**Published:** 2024-10-07

**Authors:** Zoë Vowles, Danielle C. Ashworth, Rebecca L. Barron, Frances Conti-Ramsden, Hannah Wilson, Lisa Leighton, Louise Wall, Cherrelle Walter, Jenny Myers, Lucy C. Chappell

**Affiliations:** 1 Department of Women’s and Children’s Health, School of Life Course and Population Sciences, Faculty of Life Sciences and Medicine, King’s College London, St Thomas’ Hospital, 10th Floor North Wing, London, SE7 8EH UK; 2 Maternal and Fetal Health Research Centre, Saint Mary’s Hospital, Manchester Foundation Trust, Manchester, UK; 3 Birmingham Clinical Trials Unit, University of Birmingham, Birmingham, UK; 4 Individual with Lived Experience, Manchester, UK; 5 Individual with Lived Experience, London, UK; 6 Faculty of Biology, Medicine and Health, The University of Manchester, Manchester, UK

**Keywords:** PPIE, Patient and public involvement and engagement, Stakeholder engagement, Multiple long-term conditions, Maternity research, Pregnancy

## Abstract

**Background:**

Both pregnant women and those with multiple long-term conditions are under-served groups in clinical research. Informing and improving research through patient and public involvement, including pregnant women with two or more long-term health conditions, is critical to increasing their inclusion in maternity research. Giant PANDA is a randomised controlled trial, evaluating the effect of a treatment initiation strategy with nifedipine versus labetalol on severe maternal hypertension and a composite outcome of fetal/neonatal death, or neonatal unit admission. We aimed to undertake a mixed methods study-within-a-project within the Giant PANDA trial to understand barriers and facilitators to participation, understand and optimise current representativeness of clinical trial delivery of those with multiple long-term conditions and co-create a checklist to support their inclusion in pregnancy research.

**Methods:**

We undertook online workshops with women with lived experience and hybrid workshops with healthcare professionals who look after women with multiple long-term conditions. A site audit of Giant PANDA sites provided insights into research delivery capacity and health system set-up, and how this influences inclusion. An extension to the Giant PANDA screening log captured data on multiple long-term conditions enabling analysis of the impact of these health conditions on women’s inclusion in the trial. We co-created a checklist of recommendations for those designing and recruiting to similar clinical trials.

**Results:**

Five key recommendations were identified including a need to (1) involve women with multiple long-term conditions as partners in maternity research and (2) minimise barriers that stop them from taking part through (3) designing and delivering research that is flexible in time and place (4) consider research as part of care for everyone, including those with multiple long-term conditions and (5) measure and report inclusion of those with two or more health conditions in maternity research*.* Multiple long-term conditions were not a barrier to recruitment or randomisation in the Giant PANDA trial.

**Conclusion:**

Women with multiple long-term conditions would like opportunities to find out about and participate in research which accounts for their needs. Our checklist aims to support those designing and delivering maternity research to optimise inclusion of individuals with multiple-long term conditions.

*Trial registration*: Giant PANDA: EudraCT number: 2020-003410-12, ISRCTN: 12,792,616.

**Supplementary Information:**

The online version contains supplementary material available at 10.1186/s40900-024-00634-7.

## Abstract (plain language summary)

Pregnant women with two or more long-term health problems may be less likely to be included in research. Including them in research is important to ensure we give the best care. Giant PANDA is a study comparing two medicines (nifedipine or labetalol) to manage high blood pressure in pregnancy. As part of the study, we looked at the number of women with two or more long-term health conditions included. We talked to women with experience of two or more long-term health conditions in pregnancy, and healthcare staff who look after these women. Finally, we looked at how maternity research is set up in Giant PANDA study sites. We found that women with two or more health conditions were taking part in the Giant PANDA study. Women with two or more long-term conditions would like the choice to be included in research which considers their needs. This includes being involved in the planning and ongoing support for studies. Research needs to be part of routine care, flexible, and not time consuming to help those with two or more health conditions take part. Our findings have been used to make a checklist to help plan and support studies for women and birthing people with two or more long-term health conditions.

**Supplementary Information:**

The online version contains supplementary material available at 10.1186/s40900-024-00634-7.

## Background

Health leaders have called for clinical and research communities to address issues around multimorbidity, noting that pregnancy increases the likelihood of multiple co-existing conditions [1]. One study estimates up to 18% of 19–49 year olds have multiple long-term conditions (MLTC) in the general population, with prevalence increasing from 25 to 32% from 2003 to 2016 [2]. The number of women experiencing pregnancy with pre-existing conditions such as depression, diabetes and hypertension is rising [3]. The prevalence of MLTC in pregnant women has been found to be between 16 and 24% [2, 4].

The increasing number of pregnant women with pre-existing conditions and MLTC is associated with increasing maternal age and higher body mass index and is likely to continue to rise [3, 4]. Women with multimorbidity are at increased risk of severe maternal morbidity or mortality compared to women with a single or no chronic conditions [5]. The Mothers and Babies: Reducing Risk through Audits and Confidential Enquiries across the UK (MBRRACE-UK) enquiries into maternal deaths and morbidity have elucidated the role of MLTC in adverse outcomes for pregnant women and their babies [6–8].

National Institute for Health and Care Research (NIHR) guidance recognises (among other groups) pregnant women, people with MLTC, mental health conditions, and obesity as under-served groups in research [9]. These (and other) factors may co-exist and multiply challenges to research inclusion. The latest Women’s Health Strategy for England reiterates the need to include pregnant women in clinical research [10]. Explicit factors affecting inclusivity relate to protocol inclusion/exclusion criteria, while implicit factors include issues that may affect patient participation in any research, such as the organisation of healthcare systems, the attitudes of healthcare professionals or women themselves, or the clinical study design [11].

It is critical that the research community accounts for the experiences and needs of those with lived experience of pregnancy with MLTC, through patient and public involvement. This is essential to address equity in research design and delivery, and support inclusion of pregnant women with MLTC, to ensure the evidence generated represents a real-world population. Representativeness of the study participants will increase generalisability, so that research findings can inform policy and clinical practice and ultimately, impact outcomes for women and babies [12].

The Giant PANDA Study is an ongoing multicentred randomised controlled trial, evaluating the effect of a treatment initiation strategy with nifedipine versus labetalol on severe maternal hypertension and a composite of fetal/neonatal death, or neonatal unit admission, across England and Wales. Women who decline or are unable to be randomised are offered participation in an observational part of the study involving data collection only.

The Giant PANDA researcher team have worked to promote participation of women from a variety of backgrounds (including geographical, socio-demographic, and ethnic diversity). We aimed to undertake a mixed methods study-within-a-project (SWAP) within the Giant PANDA trial, to increase understanding of the barriers and facilitators to participation, understand and optimise current representativeness of clinical trial delivery of those with multiple long-term conditions and co-create a checklist to support their inclusion in pregnancy research. This aimed to build on the INCLUDE roadmap for improving inclusion of under-served groups in clinical research with a specific focus on the ‘dynamic trial delivery’ phase onwards [9].

## Methods

### Women’s workshops

We invited women and birthing people (subsequently referred to as women or individuals) with two or more long-term conditions (MLTC) to attend two online patient and public involvement and engagement (PPIE) workshops to discuss how to make pregnancy research more inclusive and inform the ongoing delivery of the Giant PANDA trial. The two online PPIE meetings were recorded with consent, transcribed by a midwife and themes identified. Themes were identified and organised using an excel spreadsheet. These themes informed development of the checklist to support inclusion of women with MLTC in giant PANDA and future research.

To include a diverse group of women we shared information about the workshop in several ways, including through the newsletter and social media channels from the charity and patient support group Action on Pre-eclampsia. We suspected some non-human responses, therefore participants that expressed interest via social media were invited to have a call with one of the research midwives to provide further information about the project, confirm lived experience and answer any questions. We also linked with the NIHR Applied Research Collaboration for South London Maternity and Perinatal Mental Health theme PPIE network to share information about the workshop. In addition, Giant PANDA trial sites gave information to women who might be interested to participate as PPIE contributors, advertising for PPIE contributors through posters in antenatal clinics and sharing information about the workshop with women in clinical settings through postcards at sites in London and Manchester.

The first workshop focused on understanding what might help or prevent pregnant women with MLTC taking part in clinical research and suggestions to help pregnant women with MLTC find out about and participate in maternity research, to inform a checklist of recommendations to support inclusion of pregnant women with MLTC. The second workshop shared early versions of the checklist which were developed through collating themes identified from discussions during the first PPI workshop and stakeholder workshops, and sought feedback on these. The feedback was used to further develop the checklists. Workshop participants were given a £25 high street shopping voucher for expenses in keeping with PPI reimbursement guidelines [13].

### Healthcare professional workshops

Health Care Professionals working on the Giant PANDA trial were invited to participate in four regional face-to-face meetings through written, verbal and face to face engagement, with follow up of those who expressed an interest We spoke to health professional stakeholders including research midwives, research nurses, obstetricians, Principal Investigators, and Associate Principal investigators participating in the Giant PANDA trial across England and Wales. The regional meetings used a hybrid online and face-to-face format. The majority of participants joined face-to-face but the hybrid format gave an option for people to join online (using MS TEAMS) if they were unable to attend in person. Within these regional meetings we held two workshops, the first two focussed on developing a shared understanding of MLTC, explaining the purpose and rationale for the study within a project. We then used a participatory activity (the H frame evaluation exercise) to explore participants’ perceptions of how well pregnant women with two or more long-term conditions are currently included in maternity research. An H shaped diagram with a horizontal line numbered from 1 (representing not well included) to 9 (representing very well included) was drawn on flip chart paper for participants to record their views along this continuum for responses to the question. Any positive and negative factors which influence inclusion were recorded at the vertical lines at either end of the H diagram [14–16]. We asked participants to consider barriers and enabling factors from individual participants, health care/research delivery staff perspective and a health system perspective; suggestions to improve inclusion were recorded below the horizontal line.

Healthcare professionals at all participating Giant PANDA trial sites were given further opportunities to contribute to this project via an online whiteboard (Miro board). During the stakeholder workshops written feedback during the H frame activity was collated along with the online poll responses. Themes were identified. Themes from both the PPIE workshop with women and the stakeholder workshop were organised using an excel spreadsheet and formed the initial drafts of the checklist. The final two workshops focused on getting feedback on early versions of the checklist of recommendations for those designing and recruiting to clinical trials, to support inclusion of pregnant women with MLTC.

### Extended giant PANDA study screening log

The screening log is routinely collected data within the main Giant PANDA study approved by the South East Research Ethics Committee (REC) (REC reference: 20/LO/1110, IRAS reference: 284958), the Medicines and Healthcare products Regulatory Agency (MHRA), and Health Research Authority (HRA). Other aspects of this project were part of a SWAP which focussed specifically on bringing a new dimension to PPIE work to consider the lived experience of people with MLTC. The Giant PANDA screening log was extended in September 2022 to capture details of long-term conditions. Long-term conditions were categorised into 17 sub-groups (e.g. Cardiovascular, Gynaecological etc.), informed by the list of conditions developed by the MuM-PreDiCT collaboration and summarised to make data collection user friendly [17]. These data were analysed as part of the wider Equality Impact Assessment report for the Giant PANDA study, and included data from September 2022 to March 2023 (7 months total). Logistic regression models were used to assess the association (and assess for statistical significance) between MLTC status and women’s consent to and randomisation in the Giant PANDA trial.

### Site audit

We undertook a site audit to understand research delivery capacity and the health system set up, in addition to obtaining information on site-specific factors which might influence inclusion of pregnant women with MLTC. To achieve this we developed a short online site audit form (Supplementary Material 1) which was piloted at two Giant PANDA sites and revised according to feedback received. The audit form was sent by email to all 49 open Giant PANDA sites in June 2023 to be completed by the site Principal Investigator (PI), Research Matron, Clinical Trials Coordinator, or another member of the team with an overview of research delivery capacity and organisation. We raised awareness of the audit during the regular site teleconferences and through the Giant PANDA newsletter. The audit results provide context to inform the findings from the other parts of the project and recommendations.

## Results

### Women’s workshops

Fifteen women responded to the adverts, posters or postcards to express interest. Thirteen attendees (eleven different women) attended two workshops led by a research midwife and facilitated by at least one other researcher. This was 100% of women who had confirmed attendance at a workshop. Four potential PPIE participants who responded to adverts/postcards/expressed initial interest either declined attendance (due to unavailability on the planned date—one woman) or did not confirm attendance when contacted with further information and an invitation to the workshop (three women), or subsequently attend. Attendees included both pregnant and postnatal women with lived experience of MLTC in pregnancy and intentional representation across ethnicity and with both physical and mental health conditions. Participants expressed a preference that health conditions were not reported individually.

### Women’s workshop 1

At the first online workshop, five women attended and shared their ideas and experiences in response to four questions (1) what did/would make it easier or (2) what would stop you or make it more difficult for you to take part in research, (3) how could we make sure women with MLTC hear about opportunities to take part in research and finally, (4) how could we help women with MLTC take part in research during pregnancy. The responses to these discussion questions are summarised in Table 1.

**Table 1:** Women’s views on taking part in research in pregnancy with MLTC

What made it easier for you to take part in research?	What would stop you or make it difficult to take part in research?
Wanting to improve understanding of health conditions, risks in pregnancy and treatments To improve care and decision-making for future pregnancies or other women To raise awareness of health conditions in pregnancy and risks The chance to gain information and understanding for the current pregnancy	Worried about stigma from sharing information about their conditions Reluctance to share information about their condition Time commitment required to take part when juggling multiple appointments Concerns over invasive procedures e.g., blood tests and other investigations

How could we help women with MLTC hear about opportunities to take part in research?	How could we help women with MLTC take part in research?
Offer support or reassurance for people with anxiety, which may prevent participation Different members of the healthcare team discussing the study and providing information Introducing research at the pregnancy booking appointment and during pregnancy Providing information in waiting areas Providing information about research available on electronic health record apps Make taking part in research more convenient for women. Consider timing of approaching people i.e., during routine care. People may find it hard to take in information before scans or other appointments that they may feel anxious about	Provide clear and accurate information about time commitments of participation Facilitate participation flexibly (e.g., remote as well as in-person consent, offer multiple opportunities and routes for providing information) Linking research into clinical care as much as possible, e.g., linking blood tests and other investigations from care with research and vice versa Involve women with MLTC at the design stage, to get specific input about how best to support inclusion Design studies with a low burden for participation Include text or email reminders to complete questionnaires Provide written information about studies in multiple languages

### Women’s workshop 2

Eight women attended the second online workshop, including three of the five women who had attended the first workshop. Within this workshop an early version of the draft checklist of recommendations for those designing and recruiting to clinical trials was shared with women, and their feedback integrated into the next iteration of the checklist. Participants reiterated that people with MLTC want to be involved in research, with the key messages being the need to make involvement as easy as possible and integrate research with existing appointments where possible, as those with MLTC are likely to have lots of appointments during pregnancy. Involving service users with lived experience during the design of research was recommended and one person highlighted it was clear when service users had not been involved in developing studies. One participant gave an example of where research midwives in a specialist clinic integrated research into care (even when appointments were outside of the specialist clinic) which enabled her to take part. One participant mentioned that having women with lived experience of MLTC and pregnancy involved as public contributors in research also provides those women with the opportunity to meet and discuss with others going through similar situations. She suggested women find it helpful to know that others are experiencing similar issues, and that it can feel quite lonely when you have a complicated pregnancy, especially if all your friends and family had “normal” pregnancies so cannot relate to their experience.

### Healthcare professional workshops

For the London workshops, all Giant PANDA sites based in the south of England and Wales were invited. For the Manchester workshop, all Giant PANDA sites based in the north of England were invited. Table 2 shows the number and characteristics of participants.

**Table 2:** Characteristics of the healthcare professional workshop participants

Regional event no:	1a (hybrid)	1b (in person)	2b (hybrid)	2a (hybrid)
Location:	London	Manchester	London	Manchester
Month:	February	April	September	October
Number of attendees in person:	37	25	13	11
Number of sites in person:	21	15	10	10
Number of attendees online:	1	N/A	6	6
Number of sites online:	1	N/A	5	5
Total number of sites	22	15	15	15

Online polls at the first two healthcare professional workshops asked attendees their perception on the likelihood of women with MLTC (compared to those without) being offered participation in a pregnancy trial or participating in a pregnancy trial. Most healthcare professionals (58%) felt that women with MLTC (compared to those without) were less likely to be offered participation in a pregnancy trial. However, there was no consensus on the likelihood of women with MLTC participating in a pregnancy trial, compared with women without MLTC. There was a varied response to the question of how well women with MLTC are represented in research, compared with women with one condition but overall, more individuals (60%) felt women with MLTC were poorly represented.

The findings of the next stakeholder participatory activity, the H frame evaluation exercise, allowed healthcare professionals to suggest enablers and barriers that related to pregnant women/individuals who might take part in research, in addition to their ideas to improve inclusion of women with MLTC in research, all of which have been incorporated in the output of this project, described below.

Participants suggested making information sheets, posters and other materials that promote or encourage the participation of women with MLTC specifically. Approaching all women with no preconceptions, offering remote consent options; and promoting case studies to illustrate inclusion of women with MLTC studies were other ideas to improve inclusion.

At the second healthcare professional workshop we shared draft recommendations in the format of checklists for supporting inclusion in research (one focused on research design and one on research delivery). Based on the feedback we received we refined these further following each workshop into five key recommendations and one combined checklist for research design and delivery.

### Extended giant PANDA study screening log

**Fig. 1:**
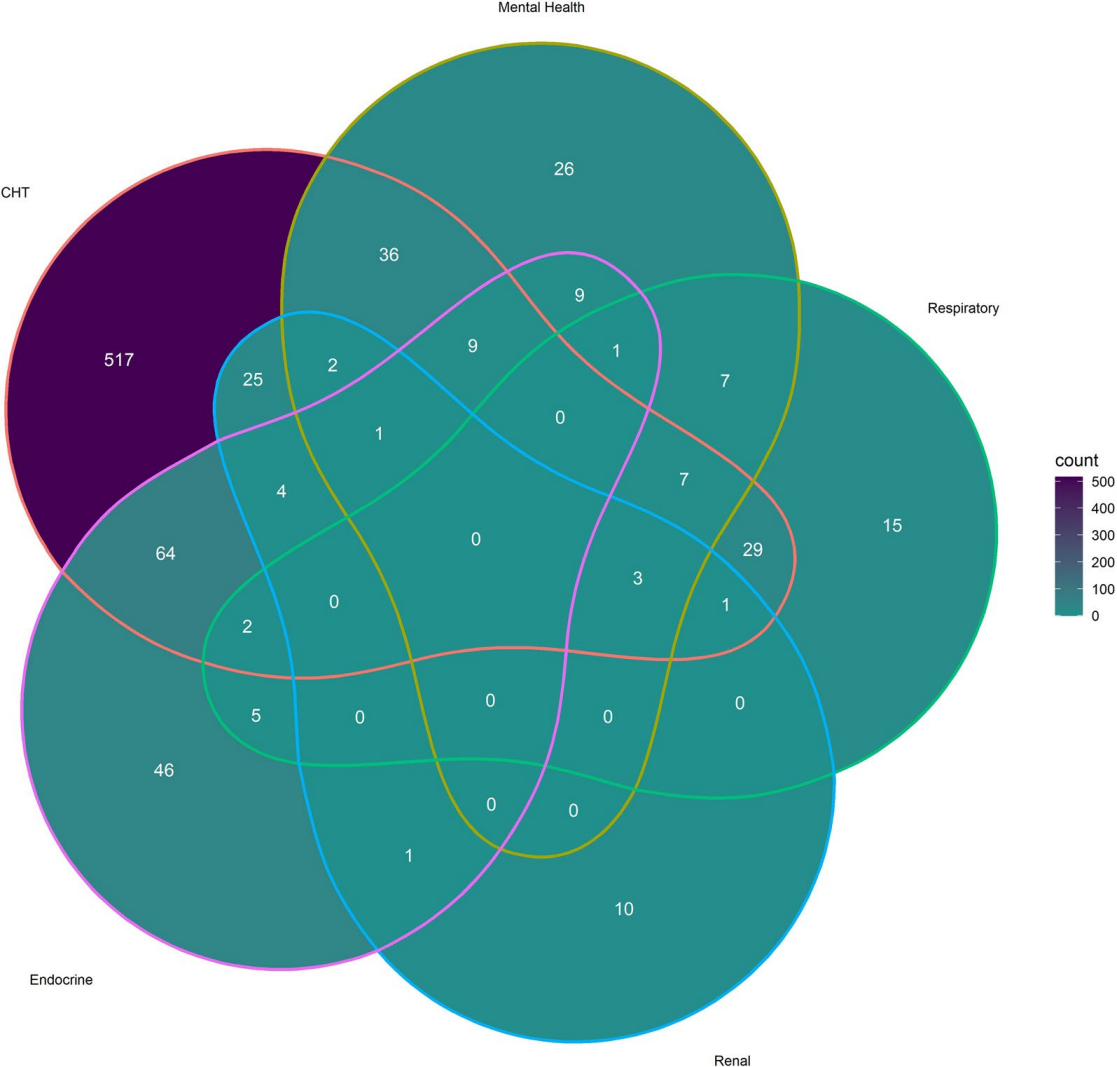
Venn diagram of overlap in chronic hypertension (CHT), mental health, respiratory, endocrine and renal conditions

A total of 1340 women eligible for Giant PANDA (observational ± randomised arm) were screened between September 2022 and March 2023, of which 286 (21.3%) had MLTCs. Chronic hypertension (CHT) 700 (52.2%), endocrine conditions 142 (10.6%), mental health conditions 101 (7.5%), respiratory conditions 70 (5.2%) and renal disease 47 (3.3%) were the most common individual long-term health conditions (LTC) reported in the extended screening log. Figure 1 shows the overlap between the most common conditions.

The association between MLTC status (comparing women with 0 or 1 LTC to those with 2 or more LTC) and recruitment to the Giant PANDA trial and recruitment to the randomised arm of the trial are shown in Tables 3 and 4.

**Table 3:** Association between MLTC status and recruitment to Giant PANDA study

MLTC status	Eligible, * n (%)	Recruited, n (%)	Odds ratio [95% CI]	*p*-value
MLTC (2 or more LTC)	286 (21.3)	212 (74.1% of eligible)	1.12 [0.83–1.51]	0.5
No MLTC (0 or 1 LTC)	1054 (78.7)	758 (71.9% of eligible)	Reference	–
Total	1340 (100.0)	970 (100.0)	–	–

*Includes randomised and observational study arms

**Table 4:** Association between MLTC status and recruitment to randomised arm of trial

MLTC status	Eligible for randomisation, n (%)	Recruited to randomised arm, n (%)	Odds ratio [95% CI]	*p*-value
MLTC (2 or more LTC)	212 (18.7)	95 (44.8% of eligible)	0.84 [0.59–1.20]	0.3
No MLTC (0 or 1 LTC)	919 (81.3)	421 (45.8% of eligible)	Reference	–
Total	1131 (100.0)	516 (100.0)	–	–

There was no statistically significant association between MLTC status and overall consent to the trial (randomised and observational arms) (Table 3). MLTC status was also not associated with odds of randomisation (Table 4).

### Site audit—health system set-up and research delivery capacity

We received responses from 24 out of 49 sites. The characteristics of sites that responded are described in Supplementary Material 2, and the findings are summarised in Supplementary Material 3. We received responses from sites of varying sizes and with a wide geographic spread. There was a large variation between the minimum and maximum average number of full-time equivalent research staff working at each site to deliver the Reproductive Health and Childbirth portfolio of studies with a mean (SD) of 4.8 (6.0) and a minimum of 0.4 and maximum of 25.5.

The wide variation in study types, clinics offered and places in which women are offered research participation across the sites is additionally demonstrated in Supplementary Material 3.

The audit form also provided a space for respondents to provide free text responses to two questions, the results of which have been summarised in Table 5. The responses to these questions have also informed the development of the checklist.

**Table 5:** summary of factors describing factors which make inclusion of women with MLTC more or less likely

What makes inclusion less likely?	What makes inclusion more likely
Less research active clinical teams and a lack of specialist clinical teams Geographically distributed maternity services e.g., multiple sites with different clinical areas in each Lack of engagement with community based clinical teams Lack of electronic health records, limiting opportunities to identify and invite eligible women to participate in research Clinician gatekeeping Inadequate dedicated research staffing and space Multiple appointments for women with MLTC, impacting time and financial considerations	Research active clinical teams Larger Trusts with diverse populations and a greater portfolio of research studies Effective utilisation of appointments for women with MLTC to offer opportunities for inclusion in studies Strong networks and relationships between clinical staff and research teams (including specialist clinical teams) Specialist and maternal medicine clinics and clinicians, including those with integrated research midwife involvement Established research delivery teams Availability of suitable studies for women with MLTC Engaged, committed Principal Investigators with an active clinical and research operational, leadership and strategic presence Wider integration of clinical and research activities (e.g., clinical staff completing Good Clinical Practice training, dual clinical and research roles, visibility of research staff in clinical areas)

### Checklist

**Fig. 2:**
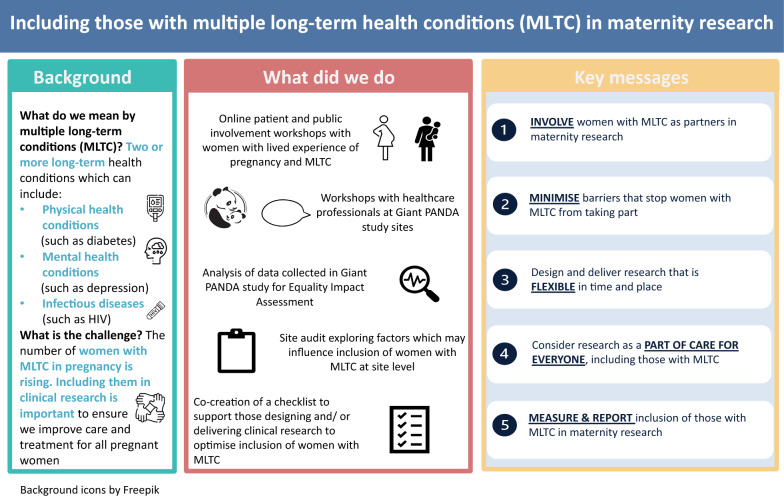
Visual Abstract

**Fig. 3:**
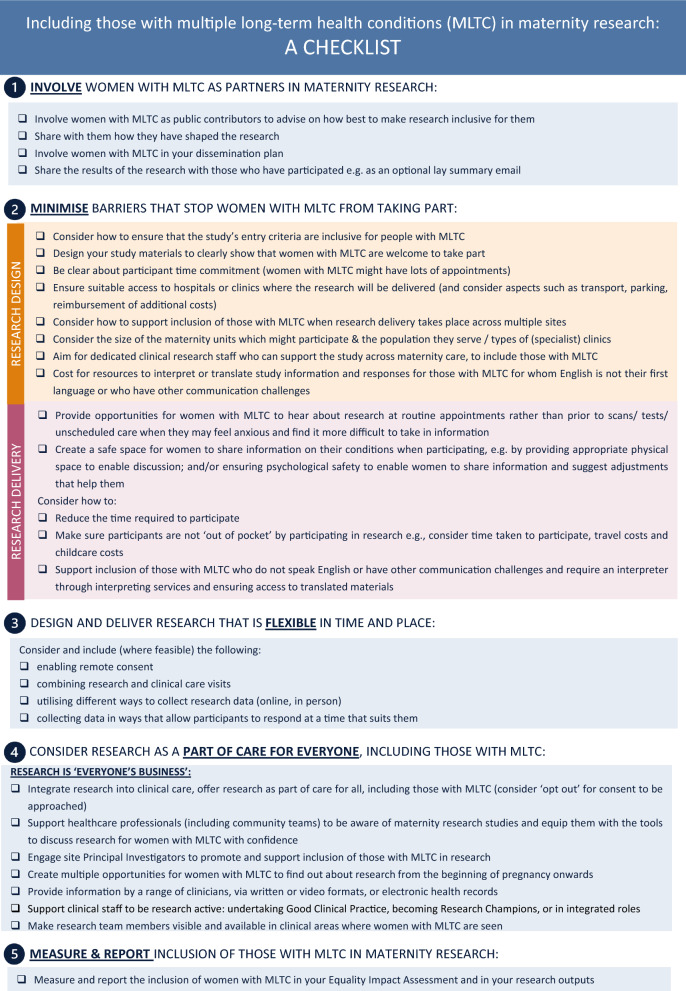
Checklist

Findings from workshops and the site audit were used to develop a visual abstract (Fig. 2) and checklist (Fig. 3) describing considerations for designing and delivering research, with the aim of supporting researchers to promote inclusion of women with MLTC in maternity research. Five key principles were identified: (1) involve women with MLTC as partners in maternity research; (2) minimise barriers that stop women with MLTC from taking part; (3) design and deliver research that is flexible in time and place; (4) consider research as part of maternity care for everyone, including those with MLTC, research is ‘everyone’s business’; and (5) measure and report inclusion of those with MLTC in maternity research. The visual abstract and checklist were designed to prompt and remind researchers of key principles to support embedding inclusive research practices for women with MLTC in maternity settings.

The checklist was designed to help researchers at the design stage and those responsible for delivery, to reflect and work through their own study and site-specific barriers and opportunities to optimise inclusion of women with MLTC in research.

## Discussion

### Key findings

We chose multi method PPIE and stakeholder engagement to address this particular challenge (as outlined in our objective). We have aimed to present our key findings as they relate to the results section. There was widespread interest and enthusiasm for inclusion of those with MLTC. Women and healthcare professionals described several opportunities for inclusion of this group in research. However, overall, slightly more healthcare professionals thought women with MLTC were less likely to be offered, or represented in, maternity research. We found that the Giant PANDA study is representative of women with two or more health conditions, suggesting that the ongoing approaches used within the trial for enabling research inclusion are successful. The findings from the PPIE and health professional workshops suggest there is further work which could be done to enhance inclusion of those with MLTC more widely in pregnancy research.

There are current initiatives for supporting inclusion of underserved groups in research. A core outcome set for studies of pregnant women with multimorbidity has also been developed [18]. However, our project was focussed on developing practical ideas for sites at a research delivery level focussed on inclusion of pregnant women with MLTC, in partnership with women and healthcare professional stakeholders. This has highlighted various areas where there is potential to make a difference to inclusion rates for women with MLTC in maternity research. Specifically inviting those with MLTC to take part in research, integration between clinical care and research, specialist clinics serving women with complex needs, and provision of suitable studies designed to account for the needs of those with multiple health conditions to enable participation of women with MLTC. Current barriers to participation include the organisation of both maternity services and clinical research delivery, clinician gatekeeping, and the time and financial implications for women with MLTC who are managing complex care and multiple appointments. The visual abstract and checklist are aimed to support those designing or delivering clinical research to evaluate how inclusive planned or existing research is of those with MLTC.

### Strengths and weaknesses

To our knowledge this is the first piece of work focussing specifically on supporting inclusion of pregnant women with MLTC in clinical research. A strength of this work is its use of multiple methods with a diverse group of participants to add to the knowledge about barriers and facilitators to research participation, for women with MLTC. The outputs have been developed with the support of significant and meaningful PPIE activities and stakeholder involvement, though they have not been formally tested or evaluated.

It is important to note that this work was aligned with a single study and involved a relatively small group of women, predominantly including those with chronic hypertension alongside another long-term condition. MLTC spans a wide range of conditions and affects women with diverse characteristics so understanding the needs and preferences of other women may be beneficial. The analysis of the trial screening log data estimated the proportion of women eligible for the Giant PANDA trial with MLTC. However, the proportion of women eligible for the trial with MLTC may have been under-estimated if clinician gatekeeping preventing participation in individuals with MLTC occurs prior to screening by research delivery staff. Reassuringly, the proportion of women with MLTC in our study (21%) was similar to that reported in the wider population [2, 4], suggesting this does not seem to have been a major issue. Clinician gatekeeping has been observed in other areas of research, such as palliative care, where potential participants are viewed as vulnerable or experiencing burden, which may be applicable to those with MLTC [19].

### Context in relation to other studies

There is a clear need to focus on inclusion of those with MLTC in pregnancy research. Recent reviews into maternal deaths and morbidity in the UK identify those with MLTC are at higher risk of experiencing the most severe adverse outcomes [7, 8]. A dose–response relationship between the number of long-term health conditions a woman has prior to pregnancy and the risk of adverse outcomes during pregnancy and the postnatal period has been suggested [5]. Widening inclusion of pregnant women with MLTC in clinical research and implementation studies is an essential part of reducing inequalities in adverse outcomes [20].

Currently MLTC status is not explicitly identified in women who are pregnant and booking for care in the UK, though past and current medical history is recorded. Reporting of pre-existing health conditions in pregnancy trials is variable and not currently standardised. Recent randomised controlled trials looking at management of long-term conditions or pregnancy complications report on the presence of comorbidities or pre-existing health conditions [21–23]. However, other studies looking at interventions to improve health during pregnancy explicitly exclude pregnant women with pre-existing health conditions [24] or do not report the presence or absence or pre-existing conditions among study participants which makes it challenging to know if research is representative of or findings applicable to those with MLTC [25]. There is a need to report the inclusion of women with MLTC in future pregnancy research.

### Implications of the findings

Much of our findings support principles of good research practice and research inclusion more broadly, which is integral to ensuring high quality and relevant research [26]. We have specifically explored and identified how this relates to the inclusion of pregnant women with MLTC. We outline ways to build on existing knowledge and recommendations for inclusion to meet the needs of pregnant women with MLTC.

From a woman’s perspective, women with MLTC value giving their time to contribute to improving knowledge, treatment, and care during pregnancy. However, to enable their participation their specific needs must be considered when designing and offering research. Involving women with lived experience of MLTC in pregnancy in the design and implementation of research studies is needed to achieve this [27].

Most medicines used to treat pre-existing health conditions have not been tested in pregnancy [3]. The 2023 MBRRACE-UK Saving Lives, Improving Mothers’ Care report identifies the need to include pregnant women in medicine and vaccine research to ensure equity for pregnant women and improve outcomes [8]. This is particularly relevant for women with MLTC who are over-represented among the women who died in pregnancy or postnatally [8]. We found that for women with MLTC, embedding a research active culture and making research part of everyday clinical practice, as advocated by the NIHR and Royal College of Physicians, is important for inclusion of women with MLTC [26, 28, 29].

From a healthcare professional or researcher perspective, embedding principles of ‘Equitable Research Design’ into the development and conduct of studies is crucial [30]. This will lead to research that is more representative and evidence that is more generalisable with greater impact on personalised care and the potential to reduce health disparities [30]. The co-created checklist produced as part of our project encourages researchers to take steps during clinical research design and delivery to enable participation and reduce barriers to inclusion for women with MLTC. This needs to be further supported by researchers using established or standardised definitions of multimorbidity and reporting of inclusion of women with MLTC in all pregnancy studies for benchmarking [5].

At a system level the opportunities identified to increase inclusion of those with MLTC were large trusts with diverse populations, and specialist services for those with complex needs or medical conditions. To avoid increasing health disparities, it is necessary to actively support inclusion in areas which currently have less research capacity or specialist services. People in communities where prevalence of long-term conditions is high may be underserved by research and there is a need to ensure research is inclusive and reaches the groups and communities where need is greatest [26]. The checklist has been designed to support inclusion across different maternity settings.

### Next steps

The checklist is designed to be used alongside existing resources to support inclusion of diverse groups such as the guidance from the NIHR INCLUDE project, the NIHR Race Equality Framework and Equality Impact Assessment Toolkit [9, 31–33]. The final checklist will be shared with all Giant PANDA sites and to support inclusion of those with MLTC in ongoing delivery of the trial. We will disseminate the checklist through clinical research networks, conferences and PPIE networks. We found embedding a research active culture and making research part of everyday clinical practice, as advocated by the NIHR and Royal College of Physicians, is key for women with MLTC [26, 28, 29].

## Conclusions

Being involved in the development, delivery and dissemination of research that accounts for their needs and having the opportunity to find out about, and participate in, clinical research as part of their maternity care is important to pregnant women with multiple long-term health conditions.

Research needs to be designed to support inclusion, minimise barriers and explicitly report inclusion of women with MLTC. These findings and the checklist developed from them will support those designing and delivering maternity clinical trials to optimise inclusion of women and birthing people with multiple-long term conditions.

## Supplementary Information

Supplementary Material 1

Supplementary Material 2

Supplementary Material 3

Supplementary Material 4

## Data Availability

No datasets were generated or analysed during the current study.

## Abbreviations

aOR: Adjusted odds ratio
CHT: Chronic hypertension
CKD: Chronic kidney disease
HRA: Health Research Authority
LTC: Long-term condition
MLTC: Multiple long-term conditions
MBRRACE-UK: Mothers and Babies: Reducing Risk through Audits and Confidential Enquiries across the UK
MHRA: Medicines and Healthcare products Regulatory Agency
NIHR: National Institute for Health and Care Research
PPIE: Patient and public involvement and engagement
RCH: Reproductive Health and Childbirth
RCT: Randomised controlled trial
SD: Standard deviation
SWAP: Study within a project
T2DM: Type 2 diabetes mellitus
