# Supplementary material for: Inclusion of people with multiple long-term conditions in pregnancy research: patient, public and stakeholder involvement and engagement in a randomised controlled trial
Source: Res Involv Engagem. 2024 Oct 7;10:101. doi: 10.1186/s40900-024-00634-7 (PMC11457413; doi:10.1186/s40900-024-00634-7)
Supplement: Supplementary file 1 — Supplementary Material 1 [file 40900_2024_634_MOESM1_ESM.pdf]

Giant PANDA Study Within A Project (SWAP)

The SWAP aims to evaluate optimisation of clinical trial delivery to maximise inclusivity and participation of pregnant women and birthing people with multiple long term conditions (two (or more) long-term conditions that need ongoing medical attention or limit daily activities).

This is part of the SWAP with the objective of evaluating health system set-up and research delivery capacity.

Thank you for participating, your time and input are very much appreciated.

* Required

1. Name of hospital/NHS trust *

2. What is the total number of reproductive health and childbirth studies you are currently actively recruiting to? *

- ◯ 0
- ◯ 1-5
- ◯ 6-10
- ◯ 11-20
- ◯ 21-30
- ◯ 31 -50
- ◯ More than 50

3. Which types of study are offered to pregnant women/birthing people in your maternity unit? **Please tick all that apply** *

- ☐ Observational
- ☐ Randomised Control Trial
- ☐ Commercial studies
- ☐ Qualitative studies
- ☐ Assisted conception/fertility studies
- ☐ Gynaecological studies
- ☐ Pre-term birth studies
- ☐ Pregnancy complications studies
- ☐ Intrapartum studies
- ☐ Postnatal studies
- ☐ Other

4. How many staff (whole time equivalent) do you have employed to deliver reproductive health and childbirth research?

(this could include Research Midwives, Research Nurses, Clinical Research Practitioners, Clinical Research Assistants or Research Assistants who can recruit to clinical trials)? *

5. Which specialist clinics are available to pregnant women in your maternity unit? **Please tick all that apply** *

- [ ] We do not offer any specialist clinics to pregnant women in our maternity unit
- [ ] Hypertension clinic
- [ ] Diabetes clinic
- [ ] Haematology clinic
- [ ] Infectious disease clinic (e.g. for women with HIV, etc)
- [ ] Cardiac
- [ ] Respiratory
- [ ] Neurology/ epilepsy
- [ ] Renal
- [ ] Obesity
- [ ] Endocrine (not diabetes)
- [ ] Gastrointestinal
- [ ] Mental health
- [ ] Alcohol and substance use
- [ ] Other

6. In which of the following locations does your maternity service offer research to women and birthing people for reproductive health and childbirth studies? **Please tick all that apply** *

- [ ] Specialist clinic(s)
- [ ] Obstetric antenatal clinic(s)
- [ ] Hospital based midwife antenatal clinic(s)
- [ ] Community based midwife antenatal clinic(s)
- [ ] Maternity Assessment Unit/Day Assessment Unit
- [ ] Gynaecology ward
- [ ] Antenatal Ward
- [ ] Labour ward/birth centre
- [ ] Postnatal ward
- [ ] Ultrasound department
- [ ] Community hub
- [ ] Woman's home
- [ ] Remotely (e.g. via telephone)
- [ ] Other

7. Is there anything specific about your maternity unit or, approach to recruitment, which means that with pregnant women with multiple long term conditions are more likely to be included within reproductive health and childbirth studies? *

8. Is there anything specific about your maternity unit, or approach to recruitment, which means that pregnant women with multiple long-term conditions are less likely to be included within reproductive health and childbirth studies? *

This content is neither created nor endorsed by Microsoft. The data you submit will be sent to the form owner.

Microsoft Forms
