# Supplementary material for: Inclusion of people with multiple long-term conditions in pregnancy research: patient, public and stakeholder involvement and engagement in a randomised controlled trial
Source: Res Involv Engagem. 2024 Oct 7;10:101. doi: 10.1186/s40900-024-00634-7 (PMC11457413; doi:10.1186/s40900-024-00634-7)
Supplement: Supplementary file 2 — Supplementary Material 2 [file 40900_2024_634_MOESM2_ESM.docx]

**Additional file two: summary of audit responses on characteristics of Giant PANDA study sites**

| **Site characteristic** | **Sites**  **N=24** |
| --- | --- |
| Births per year*  *≤2001-4000*  *4001-6000*  *>6001* | N (%)  9 (38%)  8 (33%)  7 (30%) |
| Location  *East of England*  *London*  *Midlands*  *Northeast and Yorkshire*  *Northwest*  *Southeast*  *Southwest*  *Wales* | N (%)  *2 (8)*  *5 (21)*  *4 (17)*  *2 (8)*  *3 (13)*  *2 (8)*  *3 (13)*  *3 (13)* |

*Annual average calculated using monthly data extracted from the Maternity Services Data Set for England and National Community Child Health Database for Wales.
