# Supplementary material for: Inclusion of people with multiple long-term conditions in pregnancy research: patient, public and stakeholder involvement and engagement in a randomised controlled trial
Source: Res Involv Engagem. 2024 Oct 7;10:101. doi: 10.1186/s40900-024-00634-7 (PMC11457413; doi:10.1186/s40900-024-00634-7)
Supplement: Supplementary file 3 — Supplementary Material 3 [file 40900_2024_634_MOESM3_ESM.docx]

**Additional file three – summary of audit responses on research delivery set-up at Giant PANDA study sites**

| **Giant PANDA sites responding (total)** | **n=24** |
| --- | --- |
| Number of Reproductive Health and Childbirth studies in recruitment  *1-5*  *6-10*  *11-20*  *>21* | N (%)  *8 (33)*  *6 (25)*  *7 (29)*  *3 (12)* |
| Study types offered to pregnant women (not mutually exclusive)  *Observational*  *Randomised Control Trial*  *Commercial studies*  *Qualitative studies* | N (%)  *23 (96)*  *24 (100)*  *9 (38)*  *12 (50)* |
| Study topic offered (not mutually exclusive)  *Assisted conception/fertility studies.*  *Gynaecological studies*  *Pre-term birth studies*  *Pregnancy complications studies*  *Intrapartum studies*  *Postnatal studies* | *9 (38)*  *16 (67)*  *14 (58)*  *22 (92)*  *14 (58)*  *10 (42)* |
| Specialist clinics available to pregnant women (not mutually exclusive)  *Hypertension*  *Diabetes*  *Haematology*  *Infectious disease*  *Cardiac*  *Respiratory*  *Neurology/epilepsy*  *Renal*  *Obesity*  *Endocrine (not diabetes)*  *Gastrointestinal*  *Mental health*  *Alcohol and substance use*  *Other* | N (%)  *13 (54)*  *23 (96)*  *12 (50)*  *10 (42)*  *8 (33)*  *3 (13)*  *7 (29)*  *8 (33)*  *10 (42)*  *14 (58)*  *3 (13)*  *19 (75)*  *8 (33)*  *5 (21)* |
| Location of recruitment for RCH studies (not mutually exclusive)  *Specialist clinic(s)*  *Obstetric antenatal clinic(s)*  *Hospital based midwife antenatal clinic(s)*  *Community based midwife antenatal clinic(s)*  *Maternity Assessment Unit/Day Assessment Unit*  *Gynaecology ward*  *Antenatal Ward*  *Labour ward/birth centre*  *Postnatal ward*  *Ultrasound department*  *Community hub*  *Woman's home*  *Remotely (e.g., via telephone)* | N (%)  *21 (88)*  *24 (100)*  *18 (75)*  *10 (42)*  *22 (92)*  *14 (58)*  *24 (100)*  *20 (83)*  *18 (75)*  *14 (58)*  *3 (13)*  *7 (29)*  *19 (79)* |
