# Supplementary material for: Inclusion of people with multiple long-term conditions in pregnancy research: patient, public and stakeholder involvement and engagement in a randomised controlled trial
Source: Res Involv Engagem. 2024 Oct 7;10:101. doi: 10.1186/s40900-024-00634-7 (PMC11457413; doi:10.1186/s40900-024-00634-7)
Supplement: Supplementary file 4 — Supplementary Material 4 [file 40900_2024_634_MOESM4_ESM.docx]

| **Section and topic** | **Item** | **Reported on page No** |
| --- | --- | --- |
| 1: Aim | Report the aim of PPI in the study | 6-7 |
| 2: Methods | Provide a clear description of the methods used for PPI in the study | 7-8 |
| 3: Study results | Outcomes—Report the results of PPI in the study, including both positive and negative outcomes | 10-13 |
| 4: Discussion and conclusions | Outcomes—Comment on the extent to which PPI influenced the study overall. Describe positive and negative effects | 22-23 |
| 5: Reflections/critical perspective | Comment critically on the study, reflecting on the things that went well and those that did not, so others can learn from this experience | 22 |
